# Molecular Detection of Porcine Parainfluenza Viruses 1 and 5 Using a Newly Developed Duplex Real-Time RT-PCR in South Korea

**DOI:** 10.3390/ani13040598

**Published:** 2023-02-08

**Authors:** Jong-Min Kim, Hye-Ryung Kim, Gyu-Tae Jeon, Ji-Su Baek, Oh-Deog Kwon, Choi-Kyu Park

**Affiliations:** Animal Disease Intervention Center, College of Veterinary Medicine, Kyungpook National University, Daegu 41566, Republic of Korea

**Keywords:** porcine parainfluenza virus 1, porcine parainfluenza virus 5, dqRT-PCR

## Abstract

**Simple Summary:**

In the present study, we first developed a highly specific and sensitive TaqMan probe-based duplex real-time reverse transcription polymerase chain reaction (dqRT-PCR) assay for simultaneous and differential detection of porcine parainfluenza virus 1 (PPIV1) and PPIV5 in a single reaction. The developed dqRT-PCR, with high sensitivity, specificity, and accuracy, was useful for the detection of PPIV1 and PPIV5 in clinical pig samples, and the diagnostic sensitivity was higher than that of the previous qRT-PCR for PPIV1 and consistent with that of the previous qRT-PCR for PPIV5. Using the dqRT-PCR, we demonstrated that PPIV1 and PPIV5 are co-circulating in Korean pig herds, and subsequent phylogenetic analysis suggested that genetically diverse PPIV1 strains are presented in Korean pig herds. The new dqRT-PCR developed in this study will be a valuable tool for diagnosis and further studies for PPIV1 and PPIV5.

**Abstract:**

Two species of porcine parainfluenza viruses (PPIV), PPIV1 and PPIV5, are globally distributed in pig herds and associated with porcine respiratory diseases, and a diagnostic tool for the simultaneous detection of the two viruses is required. In this study, a TaqMan probe-based duplex real-time reverse transcription polymerase chain reaction (dqRT-PCR) assay was first developed for the differential detection of PPIV1 and PPIV5 nucleocapsid protein (NP) genes in porcine clinical samples. The dqRT-PCR assay was highly sensitive, its limit of detection was approximately 10 RNA copies/reaction, it specifically amplified the targeted NP genes of PPIV1 and PPIV5 without cross-reacting with other porcine pathogens, and their clinical detection rates were 15.2% and 0.7%, respectively. The results from 441 clinical samples taken from 278 Korean domestic pig farms showed that the prevalence of PPIV1 and PPIV5 was 11.2% and 1.1%, respectively, and co-infection of both viruses was confirmed in a farm, suggesting that PPIV1 and PPIV5 are co-circulating in current Korean pig herds. Phylogenetic analysis based on the partial NP genes suggested that genetically diverse PPIV1 strains are circulating in Korean pig herds. The developed dqRT-PCR assay was found to be an accurate, reliable, and quantitative detection tool for PPIV1 and PPIV5 RNA in clinical pig samples and will be useful for etiological and epidemiological studies and the control of viral infections in the field.

## 1. Introduction

Porcine parainfluenza viruses (PPIVs) belong to the *Paramyxoviridae* family, and historically, four types have been reported to infect pigs: PPIV1, PPIV2, PPIV3, and PPIV5. PPIV1 was first discovered from nasopharyngeal and rectal samples of slaughtered pigs in Hong Kong in 2013 [1]. Subsequent studies revealed that the virus is also distributed in other Asian, American, and European countries and is associated with the induction of porcine respiratory disorders [2,3,4,5,6,7,8,9]. The virus is taxonomically classified as *porcine respirovirus 1* in the genus *Respirovirus* of the subfamily *Orthoparamyxovirinae* [10]. PPIV2 was first isolated from the fetal lung tissue of a breeding sow with porcine reproductive and respiratory syndrome (PRRS)-like symptoms in Germany in the late 1990s, and the virus was named PPIV2 due to its antigenic relationship with simian virus 5 (SV5), a prototype strain of PIV2 [11]. However, subsequent sequencing studies revealed that PPIV2 and SV5 were closely related to PIV5 strains from different hosts, and are now designated as the species *Mammalian orthorubulavirus 5* in the genus *Orthorubulavirus* of the subfamily *Rubulavirinae*, together with PIV5 strains from different hosts, including humans, pigs, dogs, cats, rodents, calves, horses, and lesser pandas [10,12,13,14,15,16,17]. PIV5 strains are commonly named according to the host from which the virus was isolated for a better scientific context; for example, human PIV5 (HPIV5), canine PIV5 (CPIV5), and porcine PIV5 (PPIV5). In pigs, PPIV5 has been recognized as a potential cause of porcine respiratory disease as it is frequently isolated from pig respiratory tracts, although there are some reports that the virus is also associated with enteric diseases [11,13,18,19]. PPIV3 was first isolated from pigs with interstitial pneumonia and encephalitis in the United States in 1981 and 1992 [20]. Subsequent molecular studies revealed that these viruses were variants of bovine parainfluenza virus type 3 (species *bovine respirovirus 3*) and were possibly transmitted from cattle to swine [21,22]. Furthermore, a serological survey carried out on swine farms in the United States showed negative results, indicating that the virus failed to acquire an active enzootic state in these pig populations [22]. Taken together, it can be summarized that two types of PPIVs, PPIV1 (*porcine respirovirus 1*) and PPIV5 (*mammalian orthorubulavirus 5*), may be prevalent in the current pig population associated with respiratory diseases, although four types of PPIVs have been reported to infect pigs in the literature.

In South Korea, PPIV5 was first identified in the lung tissues of a piglet suffering respiratory problems in 2011 [14]. A subsequent small-scale prevalence study revealed that 6 out of 28 tested pigs (21.4%) were PPIV5-positive when using a reverse transcription polymerase chain reaction (RT-PCR), and 483 out of the 515 tested porcine sera (93.8%) collected from 10 pig farms were positive for PPIV5 antibodies in a neutralization assay, suggesting that PPIV5 was highly prevalent in Korean domestic pig farms [19]. PPIV1 was first detected in pigs from 16 farms located in 7 provinces across South Korea, with a prevalence of 71.4% based on the tested oral fluid samples using the qRT-PCR assay, suggesting that this virus is also already widespread in Korean pig herds [7]. These findings suggest that these two PPIVs, PPIV1 and PPIV5, are currently prevalent in Korean pig farms. Further studies are thus required to determine the nationwide prevalence and co-infection status of these two PPIVs in Korean pig farms and develop a convenient diagnostic tool for their differential detection in clinical samples.

As virus isolation is difficult and time-consuming, RT-PCR-based assays have been developed and are now commonly used for the rapid and specific diagnosis of PPIV1 [1,2,4,6,8] and PPIV5 [13,14]. For PPIV1, gel-based conventional RT-PCR [1,2] and SYBR Green fluorescent dye-based real-time RT-PCR [1] assays described previously have been replaced by TaqMan probe-based real-time RT-PCR (qRT-PCR) assays owing to their high specificity and sensitivity, rapidity, and quantitative capabilities [4,6,8], whereas, for PPIV5, gel-based cRT-PCR assays [13,14] have been developed, but qRT-PCR has not yet been reported.

Considering that both PPIV1 and PPIV5 are globally distributed and associated with porcine respiratory diseases, a duplex qRT-PCR (dqRT-PCR) assay that can differentially diagnose the two viruses is urgently required. To address this issue, we developed a dqRT-PCR assay based on TaqMan probe technology for the differential diagnosis of PPIV1 and PPIV5 from porcine clinical samples in the present study. Furthermore, we investigated the prevalence of viral infections in Korean domestic pig farms using the newly developed dqRT-PCR assay.

## 2. Materials and Methods

### 2.1. Viruses and Clinical Samples

PPIV1 (KPPIV1-2201 strain) and PPIV5 (KPPIV5-2201 strain) Korean field strains (GenBank accession numbers ON457669 and OP734281, respectively) were obtained and used to develop and optimize the dqRT-PCR assay [7]. To evaluate the specificity of the assay, seven porcine viral pathogens, including porcine circovirus 2 (PCV2, PCK0201 strain), PCV3 (PCK3-1701 strain), type 1 porcine reproductive and respiratory syndrome virus (PRRSV, Lelystad virus), type 2 PRRSV (LMY strain), swine influenza virus (SIV, VDS1 strain), classical swine fever virus (LOM strain), and porcine parvovirus (NADL-2 strain) were also obtained from previous studies [23], and two porcine-origin cell cultures not infected with PPIV1 and PPIV5 (ST cell and PK-15 cell) were used as negative controls (Table 1). All pathogen samples were allocated and stored at –80 °C until use.

To clinically evaluate the dqRT-PCR, 441 pig samples (168 lungs, 120 oral fluids, and 153 sera) were collected from 278 pig farms that reported respiratory problems in 2019 and 2022 in South Korea. Lung tissue samples were homogenized and diluted 10-fold with phosphate-buffered saline (0.1 M, pH 7.4). All samples were centrifuged at 10,000× *g* (Hanil, Republic of Korea) for 10 min at 4 °C. Using a TANBead nucleic acid extraction kit with a fully automated magnetic bead operating platform (Taiwan Advanced Nanotech Inc., Taoyuan, Taiwan), viral RNA was extracted from each sample (200 μL) and eluted into the elution buffer (100 μL), according to the manufacturer’s instructions. All nucleic acid samples were stored at –80 °C.

### 2.2. Primers and Probes for dqRT-PCR

Based on previous RT-PCR assays targeting the nucleocapsid protein (NP) genes of PPIV1 [6,8] and PPIV5 [14], primers and probe sets were designed for the dqRT-PCR for the differential detection of the two viruses using NP gene sequences for 23 PPIV1 strains and 58 PPIV5 strains, which were retrieved from GenBank (Supplementary Tables S1 and S2). Given the broad host range of PIV5 and high genetic homology between strains isolated from different hosts [12,24], the PIV5 NP gene sequences from different hosts (26 swine strains, 2 bovine strains, 14 canine strains, and 16 other host strains) were collected and used to design the PPIV5-specific primers and probe set (Supplementary Table S2). Conserved nucleotide sequences within the NP gene of PPIV1 and PPIV5 were identified using the multiple alignments tool of the BioEdit Sequence Alignment Editor program (http://www.mbio.ncsu.edu/BioEdit/bioedit.html, accessed on 11 November 2022). Based on the conserved regions of the NP gene sequences, two pairs of primers and probes were designed to specifically detect PPIV1 and PPIV5 using Primer Express software (version 3.0) (Applied Biosystems, Foster City, CA, USA). To check the specificity of the designed primers and probe sets for PPIV1 (P1-NF, P1-NR, and P1-NP) and PPIV-5 (P5-NF, P5-NR, and P5-NP), the targeted sequences were aligned with the NP gene sequences of the PPIV1 and PPIV5 strains. The results showed that the designed primer and probe sequences for PPIV1 and PPIV5 completely matched with the corresponding viral sequences (Supplementary Figures S1 and S2).

Furthermore, the potential cross-reactivity of the primers and probes for PPIV1 and PPIV5 was confirmed using random nucleotide sequences and BLAST (http://www.ncbi.nlm.nih.gov/BLAST/, accessed on 13 November 2022). For the accurate differential detection of PPIV1 and PPIV5 using the dqRT-PCR, reporter dyes that distinct or minimally overlap with the fluorescence spectra must be used to label the sequence-specific probes [25]. For the simultaneous and differential detection of the NP genes in a single reaction in this investigation, probes were differently labeled at the 5′ and 3′ ends with 6-carboxyfluorescein (FAM) and Black Hole Quencher 1 (BHQ1) for PPIV1, and 6-tetramethylindo(di)-carbocyanines 5 (Cy5) and Black Hole Quencher 3 (BHQ3) for PPIV5, according to the manufacturer’s guidelines (BIONICS, Daejeon, Korea), respectively (Table 2).

### 2.3. Reference Gene Construction for dqRT-PCR

The partial NP genes for PPIV1 or PPIV5 spanning the amplified region of each virus using dqRT-PCR and the reference qRT-PCR assays were amplified using RT-PCR from the PPIV1 KPPIV1-2201 and PPIV5 KPPIV5-2201 strains with a pair of primers (forward, 5′- CCTCAAAGAGACAGATCAGG-3′ and reverse, 5′-TACGGCTGAATAACCATTCTG-3′ for PPIV1, and forward, 5′-TTCTGTGGTCTTTTCATAGT-3′ and reverse, 5′-CCATAAGGTTCCTGCCTA-3′ for PPIV5), which were designed based on their NP gene sequences, respectively. Reverse transcription of the cDNA was performed using a commercial kit (PrimeScript™ 1st strand cDNA Synthesis Kit, Takara, Shiga, Japan). Then, PCR was performed with a commercial kit (TaKaRa Ex Taq^®^; Takara, Shiga, Japan) in 50 μL reaction mixtures containing 5 μL of 10× Ex Taq buffer, 0.25 μL of TaKaRa Ex Taq, 4 μL of dNTP mixture, 0.2 μM of each primer, and 5 μL of PPIV cDNA as a template, according to the manufacturer’s instructions. Amplification was carried out in a thermal cycler (Applied Biosystems, Foster City, CA, USA) under the following conditions: initial denaturation at 98 ℃ for 1 min, followed by 35 cycles at 98 °C for 10 s, 58 °C for 30 s, and 72 °C for 1 min, and a final extension at 72 °C for 5 min. The amplified 460 bp NP gene of PPIV1 or 1705 bp NP gene of PPIV5 was inserted into the pTOP TA V2 vector (Enzynomics, Daejeon, Korea). The recombinant plasmid DNA of PPIV1 and PPIV5 NP genes were linearized with EcoRI (TaKaRa Bio, Kusatsu, Japan) and transcribed in vitro with RiboMAX Large Scale RNA Production System-T7 (Promega, Fitchburg, Wisconsin, USA) following the manufacturer’s recommendations. The concentration of RNA transcripts was determined using a NanoDrop spectrophotometer (Thermo Fisher Scientific, Waltham, MA, USA), and the copy numbers of the RNA transcript were quantified as follows: [copies/reaction = concentration of plasmid (g/μL)/[(plasmid length × 340) × (6.022 × 10^23^)]. The RNA transcripts were serially diluted 10-fold (10^7^ to 10^0^ copies/μL) and stored at –80 °C until further use.

### 2.4. Reference qRT-PCR Assays

To comparatively evaluate the diagnostic performance of the dqRT-PCR assay, the qRT-PCR assays for PPIV1 [8] and PPIV5 [26] were performed according to a previously described protocol with some modifications. The qRT-PCR assay for PPIV1 was performed with the PPIV1 NP gene-specific primers and probe set (Table 2) using a commercial one-step real-time RT-PCR kit (RealHelix™ qRT-PCR Kit [v4], NanoHelix, Daejeon, Korea). In brief, qRT-PCR was performed in a 25 μL reaction mixture containing 12.5 μL of 2× reaction buffer, 1 μL of 25× enzyme mix, 0.4 μM of each PPIV primer and 0.2 μM of the probe, 5 μL of RNA, and 4 μL of nuclease-free water. The reaction was carried out in a CFX96 Touch™ Real-Time PCR Detection System (Bio-Rad, Hercules, CA, USA) under the following conditions: reverse transcription at 50 °C for 30 min; initial denaturation at 95 °C for 15 min; 40 cycles of denaturation at 95 °C for 15 s; and annealing and extension at 55 °C for 60 s, according to the manufacturer’s instructions. At the end of each annealing step, real-time fluorescence signals generated by the FAM-labeled probe were measured in ongoing reactions. To interpret the qRT-PCR results, samples producing a cycle threshold (Ct) ≤ 40 were considered positive. When no Ct values were observed during the 40 amplification cycles, the sample was considered negative, as described previously [8]. Although qRT-PCR assays have been described for the detection of PIV5 strains from different animal origins, including canine [26] and bovine [27], no qRT-PCR assay has been reported for PPIV5. However, previous studies showed that the qRT-PCR assay developed for CPIV5 could successfully detect PIV5 from other animal origins as the genetic diversity of the PIV5 strains was remarkably low, regardless of host, year of isolation, or geographical origin [10,17,24]. Therefore, Dong’s previously described qRT-PCR assay for canines [26] was selected as a reference assay for PPIV5 in this study. As expected, the sequences of the primers and probes were perfectly matched with the PPIV5 NP gene sequences collected in this study. The qRT-PCR assay for PPIV5 was performed with the NP gene-specific primers and probe set (Table 2) using a commercial one-step real-time RT-PCR kit (RealHelix™ qRT-PCR Kit [v4], NanoHelix, Daejeon, Korea). In brief, qRT-PCR was performed in a 25 μL reaction mixture containing 12.5 μL of 2× reaction buffer, 1 μL of 25× enzyme mix, 0.5 μM of each PPIV primer, and 0.1 μM of probe, 5 μL of RNA, and 4 μL of water. The reaction was carried out in a CFX96 Touch™ Real-Time PCR Detection System (Bio-Rad, Hercules, CA, USA) under the same reaction conditions as mentioned above. At the end of each annealing step, real-time fluorescence signals generated by the FAM-labeled probe were measured in ongoing reactions. To interpret the qRT-PCR results, samples with Ct ≤ 40 were considered positive. When no Ct values were observed during the 40-amplification cycle, the sample was considered negative, as described previously [26].

### 2.5. Optimization of dqRT-PCR Conditions

Before optimization of the dqRT-PCR, a monoplex qRT-PCR assay with PPIV1 or PPIV5-specific primers and a probe set was optimized using a commercial real-time qRT-PCR kit (RealHelix™ qRT-PCR Kit [v4], NanoHelix, Daejeon, Korea) and CFX96 Touch™ Real-Time PCR Detection System (Bio-Rad, Hercules, CA, USA). The 25 μL reaction mixture containing 12.5 μL of 2× Reaction Buffer, 1 μL of 25× enzyme mix, 0.4 μM of each primer, 0.2 μM probe, and 5 μL PPIV1 or PPIV5 RNA template (10^6^ to 10^0^ copies/reaction) was prepared according to the manufacturer’s instructions. The concentrations of primers and probe were optimized by testing different combinations of primer concentrations, while other reaction components were maintained identical to those used in the monoplex qRT-PCR. Both monoplex qRT-PCR and dqRT-PCR conditions were the same, as follows: 30 min at 50 °C for reverse transcription, 15 min at 95 °C for initial denaturation, followed by 40 cycles of 95 °C for 15 s and 60 °C for 60 s for amplification. At the end of each annealing step, FAM (PPIV1) and Cy5 (PPIV5) fluorescence signals were obtained. To interpret the monoplex and dqRT-PCR results, samples producing a Ct ≤ 37 were considered positive, while those with Ct > 37 were considered negative, according to previously described guidelines [28].

### 2.6. Specificity and Sensitivity of dqRT-PCR

To evaluate the assay’s specificity, the dqRT-PCR assay was performed with the total nucleic acids extracted from seven viral samples and two non-infected porcine-origin cell cultures described in Table 1. The sensitivity of the dqRT-PCR and its corresponding monoplex assay for PPIV1 and PPIV5 were determined in triplicate using serial dilutions (10^6^ to 10^0^ copies/reaction) for each standard RNA of PPIV1 and PPIV5. For data analysis, a standard curve based on the Ct values of the 10-fold dilutions of PPIV1 or PPIV5 standard RNAs (from 10^6^ to 10^0^ copies/reaction) was created using CFX96 Touch™ Real-Time PCR Detection software (Bio-Rad, Hercules, CA, USA). The correlation coefficient (*R*^2^) of the standard curve, the standard deviations of the results, and the PPIV1 or PPIV5 RNA copy numbers in the samples were calculated by the detection software based on the standard curves. The efficiency of the assay was determined using the previously described calculation [29].

### 2.7. dqRT-PCR Precision

The intra-assay precision (repeatability) and inter-assay precision (reproducibility) of the dqRT-PCR assay for PPIV1 and PPIV5 were evaluated by testing three different concentrations (high, medium, and low) of each viral standard gene. The concentrations of the NP genes for PPIV1 or PPIV5 were 10^6^, 10^4^, and 10^2^ copies/reaction. Using each dilution, the intra-assay variability was analyzed in triplicate on the same day, whereas the inter-assay variability was analyzed in six independent experiments performed by two operators on different days according to the MIQE guidelines [29]. The coefficient of variation (CV) for the Ct values was determined based on the intra- or inter-assay results and expressed as a percentage of the mean value together with the standard deviation values.

### 2.8. Clinical Evaluation of dqRT-PCR

To clinically evaluate the dqRT-PCR assay, 441 pig samples (168 lungs, 120 oral fluids, and 153 sera) were collected from farms across South Korea where the pigs had respiratory problems in 2019 and 2022, tested using the newly developed dqRT-PCR assay, and the results were compared with those of the reference qRT-PCR assays for PPIV1 and PPIV5 [8,26]. The concordance between dqRT-PCR and reference PPIV1 or PPIV5 qRT-PCR was analyzed using Cohen’s kappa statistics at a 95% confidence interval (CI). The calculated kappa coefficient value (κ) was interpreted as κ < 0.20 = slight agreement, 0.21–0.40 = fair agreement, 0.41–0.60 = moderate agreement, 0.61–0.80 = substantial agreement, and 0.81–1.0 = almost perfect agreement [30]. To determine whether the cause of the discrepancy between the assays was due to a mismatch between the primers/probe and the viral target gene sequences, partial NP genes of the virus were amplified using RNAs obtained from discordant or concordant clinical samples and sequenced using Sanger’s method by a commercial sequencing company (BIONICS, Daejeon, Korea). To further analyze the discordant samples, SYBR Green-based qRT-PCR was conducted using the same reaction conditions as Schuele’s qRT-PCR for PPIV1, except that the probe was removed and SYBR Green intercalating dye was added to the reaction mixture. Furthermore, phylogenetic trees were constructed with the partial NP gene sequences and previously reported PPIV1 sequences using the Maximum Likelihood method with MEGA X software (version 10.2.4) [31,32]. The standard for lineage classification was based on the results of a previous report [7]. The tree was subjected to bootstrap analysis with 1000 replicates to determine the percentage reliability values for each internal node.

### 2.9. Prevalence of PPIV1 and PPIV5 in Korean Pig Herds

Based on the diagnostic results of the dqRT-PCR for the clinical samples, the farm-level and individual pig sample-level prevalence values were determined, as was the co-infection status for PPIV1 and PPIV5 in Korean pig herds.

## 3. Results

### 3.1. Interpretation of the dqRT-PCR Assay

**Figure 1:**
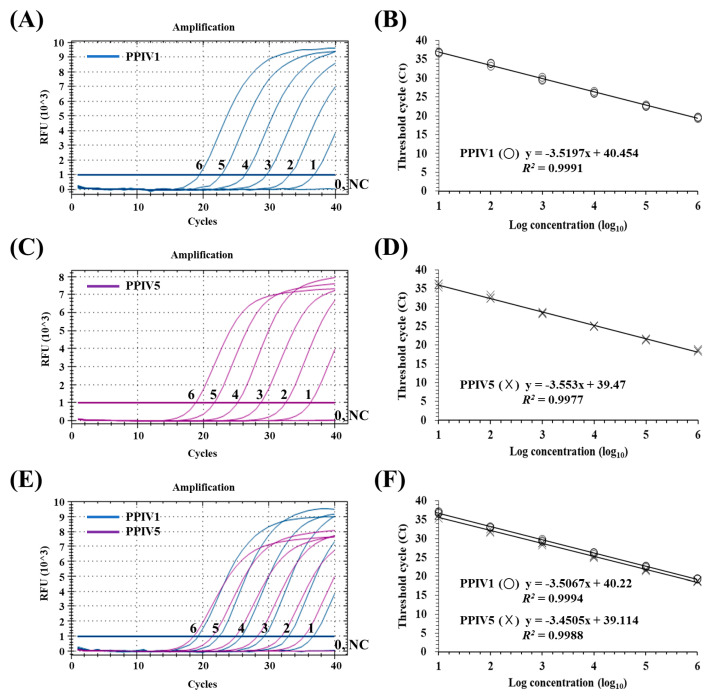
Limit of detection (LOD) and standard curves for the monoplex and duplex real-time reverse transcription polymerase chain reactions (dqRT-PCR) for porcine parainfluenza virus 1 (PPIV1) and PPIV5. LODs and standard curves of monoplex qRT-PCR for PPIV1 (**A**,**B**) and PPIV5 (**C**,**D**), and dqRT-PCR for PPIV1 and PPIV5 (**E**,**F**). Lines 6–0 are 10-fold serial dilutions of the PPIV1 and PPIV5 standard RNAs (10^6^–10^0^ copies, respectively). Standard curves of the assays were generated using 10-fold serial dilutions for PPIV1 and PPIV5 standard RNA (10^6^–10^0^ copies). The coefficient of determination (*R*^2^) and regression curve equations (y) were evaluated using the CFX Manager Software (Bio-Rad, Hercules, CA, USA).

The fluorescent FAM signals for PPIV1 or Cy5 for PPIV5 were generated by each of the corresponding monoplex qRT-PCRs (Figure 1A,C). To detect the NP genes of PPIV1 and PPIV5 simultaneously and differentially in a single reaction tube, both sets of primers and probes for dqRT-PCR were used with the same RT-PCR conditions in a multiplex format. The results of the dqRT-PCR using the optimized primer concentration (0.4 μM of each primer and 0.2 μM of each probe for PPIV1 and PPIV5) showed that the FAM signal for PPIV1 and Cy5 signal for PPIV5 could be detected simultaneously using the assay (Figure 1E). These results showed that the dqRT-PCR could successfully amplify the NP genes of both viruses in a single reaction while avoiding spurious amplification and significant crosstalk between both fluorescent reporter dyes. The efficiency of the dqRT-PCR for PPIV1 and PPIV5 was 92.8% and 94.9%, respectively, which was comparable to each of the corresponding monoplex qRT-PCRs for PPIV1 (92.4%) and PPIV5 (91.2%), as shown in Figure 1.

### 3.2. Specificity of the dqRT-PCR Assay

Each set of primers and probes for PPIV1 and PPIV5 only detected the RNA corresponding to their respective viruses. Negative results were obtained with the other seven swine pathogens and two cell cultures (Table 1). As expected, the NP genes of PPIV1 and PPIV5 were co-amplified from a mixed sample of PPIV1 and PPIV5 (Figure 1E). The results showed that the dqRT-PCR assay with two sets of primers and probes could specifically and differentially detect PPIV1 and PPIV5.

### 3.3. Sensitivity of the dqRT-PCR Assay

In terms of NP gene copy number, the limit of detection (LOD) for the dqRT-PCR was below 10 gene copies/reaction for PPIV1 and PPIV5, which was similar to the LODs for each of the monoplex qRT-PCRs (Figure 1A,C,E). Standard curves for targeted genes were generated by plotting their Ct values against their dilution factors to determine PCR efficiency and the linearity of the reaction. High correlation (*R*^2^ > 0.99) between the Ct values and the dilution factors was found for the monoplex qRT-PCR and dqRT-PCR assays (Figure 1B,D,F).

### 3.4. Precision of the dqRT-PCR Assay

To assess the precision of the dqRT-PCR, three concentrations (high, medium, and low) for each standard RNA were tested in triplicate in six independent runs performed by two experimenters on different days. The coefficients of variation within the runs (intra-assay variability) ranged from 0.09 to 0.4% for PPIV1 and from 0.29 to 0.55% for PPIV5, respectively. The inter-assay variability ranged from 0.53 to 0.67% for PPIV1 and from 0.57 to 1.44% for PPIV5, respectively (Table 3).

### 3.5. Clinical Evaluation of the dqRT-PCR Assay

To evaluate the clinical diagnostic performance of the developed dqRT-PCR assay, 441 clinical samples (168 lungs, 120 oral fluids, and 153 sera) were tested, and the results were compared with those of previously described qRT-PCR methods for PPIV1 and PPIV5 [8,26]. The detection rates for PPIV1 and PPIV5 using the dqRT-PCR assay were 15.2% (67/441) and 0.7% (3/441), whereas the detection rates for PPIV1 and PPIV5 using previously described qRT-PCR assays were 10.9% (48/441) and 0.7% (3/441), respectively (Table 4). The results of the new dqRT-PCR assay for PPIV5 were in perfect agreement with those of the previous qRT-PCR assay, regardless of the tested sample types (κ = 1.0). However, for PPIV1, 19 more clinical samples (1 lung and 18 oral fluid samples) were determined as PPIV1-positive using the new dqRT-PCR assay when compared with the previous PPIV1 qRT-PCR assay. The positive, negative, or overall agreement between both dqRT-PCR and qRT-PCR assays for PPIV1 was 100% (48/48), 95.2% (374/393), and 95.7% (422/441), respectively, resulting in kappa values (95% CI) of 0.81 (0.67–0.95). According to the sample type, the detection rate of PPIV1 was highest in oral fluids at 50.0%, and 7% in lung samples, but PPIV1 was not detected in serum samples. The detection rate of PPIV5, however, was 1.2% in lung samples and 0.8% in oral fluids, but PPIV5 was not detected in serum samples. Co-infection of PPIV1 and PPIV5 was confirmed in only one oral fluid sample (Table 4). These clinical evaluations demonstrate that the clinical diagnostic sensitivity of the new dqRT-PCR assay was higher than that of the previous qRT-PCR for PPIV1 and comparable to that of the previous qRT-PCR for PPIV5.

**Figure 2:**
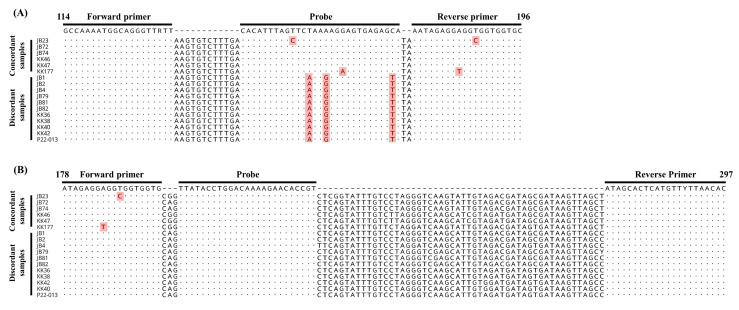
Alignments of the partial nucleocapsid protein gene sequences for porcine parainfluenza virus 1 (PPIV1) obtained from eleven discordant and six concordant clinical samples. Primers and probe binding sites of Schuele’s real-time reverse transcription polymerase chain reaction (qRT-PCR) (**A**) and the newly developed duplex qRT-PCR (**B**) are indicated by black arrows and lines. A dot indicates the same base, and a letter with a red background indicates a mismatched base.

To elucidate the cause of the discrepancy between the results of the two assays for PPIV1, partial NP genes were amplified from 19 discordant samples and another 6 concordant samples, and 17 sequences from 11 discordant and 6 concordant samples with relatively low Ct values were successfully analyzed (Table 5) and aligned to assess if there was any mismatch in the primers and probe binding region (Figure 2). The results revealed that there were no mismatches in the forward and reverse primer binding regions, but there were three mismatches in the probe binding region of the NP gene sequences obtained from 11 discordant samples where the 13th, 16th, and 28th bases of the probes altered from T to A, A to G, and C to T in the NP gene sequences, respectively. For sequences obtained from six concordant samples, no mismatch was found in the sequences of four samples, but one mismatch was found in each probe and the forward primer binding sites of the sequences of two samples (JB23 and KK177). To further confirm whether the negative results of Schuele’s qRT-PCR were due to mismatches in the probe binding site, the 19 discordant samples were retested using SYBR Green-based qRT-PCR under the same reaction conditions as Schuele’s qRT-PCR, except that the probe was excluded and SYBR Green intercalating dye was added to the reaction mixture. Nineteen discordant samples were identified as PPIV1-positive by the SYBR Green-based qRT-PCR with Ct values similar to those generated by the new dqRT-PCR assay (Table 5), indicating that results of the Schuele’s qRT-PCR for the nineteen discordant samples were false negatives, which were caused by the mismatches in the probe binding region.

### 3.6. Genetic Diversity of PPIV1 Strains Based on the NP Gene

**Figure 3:**
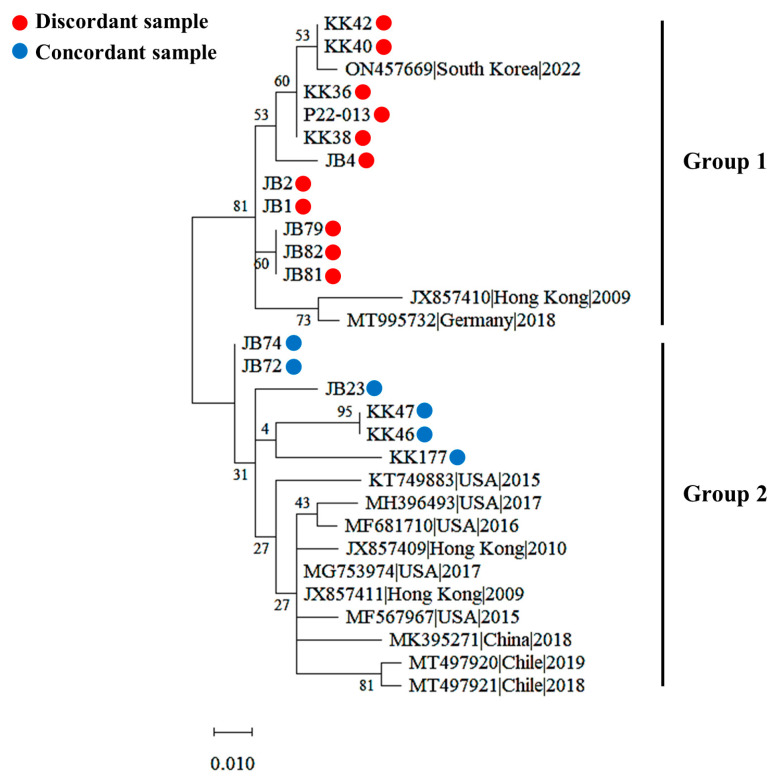
Phylogenetic tree for 30 partial nucleocapsid protein gene (184 bp) sequences from porcine parainfluenza virus 1 (PPIV1), including 17 sequences obtained in this study and 13 previously reported PPIV1 strains from GenBank. The Maximum Likelihood trees were inferred with 1000 rapid bootstrap replicates in MEGA X software, version 10.2.4. Red and blue circles indicate discordant and concordant clinical samples, respectively.

Phylogenetic analyses of the partial NP gene (184 bp) sequences derived from 17 discordant and concordant samples were clustered into 2 different groups, respectively (Figure 3). Eleven sequences from discordant samples were clustered into group 2, along with three PPIV1 strains from Hong Kong (JX857410), Germany (MT995732), and South Korea (ON475669) that were classified as lineage 1 based on the complete viral genomic sequence in the previous study [7], whereas six sequences from discordant samples were clustered in group 2, along with ten PPIV1 strains from Hong Kong (JX857409 and JX857411), the USA (KT749883, MG753974, MF567967, MF681710, and MH396493), China (MK395271), and Chile (MT497920 and MT497921) that were classified as lineage 2 in a previous study [7]. These results suggest that at least two genetically distinct groups of PPIV1 strains in South Korea are based on NP gene diversity.

### 3.7. Prevalence of PPIV1 and PPIV5 in Korean Pig Herds 

Based on the diagnostic results of the dqRT-PCR for all clinical samples in this study, farm-level and individual-pig-level prevalence of PPIV1 and PPIV5 were determined to be 11.2% (31/278) and 15.2% (67/441), or 1.1% (3/278) and 0.7% (3/441), respectively. The co-infection rates for individual pig samples and at the farm level were 0.4% (1/278) and 0.2% (1/441), respectively (Table 6).

## 4. Discussion

The paramyxoviruses PPIV1 and PPIV5 are distributed globally in pig herds and associated with porcine respiratory diseases [2,3,4,5,6,7,8,9,13,18,19]. As both viruses may be prevalent in pig herds at the same time, a dqRT-PCR assay is urgently required to simultaneously and differentially detect both viruses in suspected porcine clinical samples. However, to the best of our knowledge, such a dqRT-PCR method has not yet been developed. To address this, we have successfully developed a sensitive, specific, and reliable dqRT-PCR assay which can detect NP genes from PPIV1 and PPIV5 in a single reaction. In this study, TaqMan probe technology was used for developing a dqRT-PCR assay owing to the disadvantages associated with SYBR Green-based qRT-PCR, such as potential non-specific signals by the SYBR Green intercalating dye and the need for additional melting curve analysis for discrimination of co-amplified targets [33]. Considering the aim of this study, i.e., focusing on specific and differential detection of two PPIVs in a single reaction, the TaqMan probe-based assay was preferred to the SYBR Green-based method, which is more reliable and robust for detecting PPIV1 and PPIV5 in clinical samples.

It is well known that the selection of a target gene and the identification of conserved region within the selected gene sequence are crucial factors for optimal design of real-time RT-PCR assays. Both PPIV1 and PPIV5 have non-segmented negative-strand RNA genomes, and the NP gene is located at the 3′ end of the genome and has the largest number of transcripts as a result of gradient transcription in paramyxoviruses; this is because the promoter-proximal genes at the 3′ end of the genome are transcribed more efficiently than the promoter-distal genes at the 5′ end of the genome [34]. Furthermore, the NP gene is also one of the most conserved genes in the paramyxovirus genome [18]. Consequently, many previously reported qRT-PCR assays for paramyxoviruses have designed their primers and probes using the conserved NP gene sequences [6,8,26,35,36]. Likewise, in the present study, they were also selected as the target genes for the primers and probes in the dqRT-PCR assay (Table 2). Each of the monoplex qRT-PCRs showed that the newly designed primers and probe sets for PPIV1 and PPIV5 specifically amplified and detected their NP genes but did not detect those of the other porcine pathogens, negative tissue samples, or the negative control, suggesting that each of the primers and probe sets is highly specific to their respective PPIV1 or PPIV5 NP gene (Table 1). Furthermore, the dqRT-PCR using two sets of primers and probes for PPIV1 and PPIV5 simultaneously amplified the NP genes of two viruses and differentially detected them using different report dyes in a reaction (Figure 1). The LODs for the developed dqRT-PCR assay for PPIV1 and PPIV5 were below 10 copies/reaction, which is comparable to those for their corresponding monoplex qRT-PCR assays (Figure 1). Considering that the LODs for the recently described qRT-PCR assays for PPIV1 and CPIV5 were approximately 10 copies/reaction [4,26], the sensitivity of the dqRT-PCR assay was found to be sufficient for the detection of PPIV1 and PPIV5 in clinical samples; provided that the viruses are present at more than 10 copies/sample, which was demonstrated in clinical evaluations of the assay in this study.

In South Korea, PPIV1 was most recently identified from respiratory diseased pigs in 2022, and 6.3% (5/80) of lung samples and 71.4% (30/42) of oral fluid samples were found to be PPIV1-positive using qRT-PCR [7], whereas PPIV5 was isolated from the lung tissue of a piglet in 2011 [14] and a subsequent small-scale prevalence study on 10 pig farms showed that 93.8% (483/515) of tested pigs were seropositive for PPIV5 [19]. However, further studies are required to better understand the prevalence of the viral infections in Korean pig herds owing to the limited number of farm investigations that have been reported. Therefore, in this study, 441 pig samples (168 lungs, 120 oral fluids, and 153 sera) were collected from 278 farms across South Korea that had pigs suffering from respiratory problems. They were tested using the newly developed dqRT-PCR assay to better understand the prevalence of the viral infection, and the results were compared with previously described qRT-PCR assays [8,26]. In the clinical evaluation, the diagnostic sensitivity of the new dqRT-PCR for PPIV1 (15.2%) and PPIV5 (0.7%) was higher than that of Schuele’s qRT-PCR (10.9%) and consistent with that of Dong’s qRT-PCR (0.7%), respectively (Table 4). For PPIV1, 19 clinical samples (1 lung and 18 oral fluids) tested negative for PPIV1 using Schuele’s qRT-PCR but were determined to be positive for PPIV1 using the newly developed dqRT-PCR. However, these 19 discordant samples were confirmed as PPIV1-positive using the modified Schuele’s qRT-PCR employing SYBR Green intercalating dye instead of the probe and showed similar Ct values with those of the new dqRT-PCR (Table 5), indicating that these discordant samples were true positives and that the false-negative results were caused by the probe used in the assay. Previous studies reported that sequence mismatches between the target gene and the primers and probe may decrease the sensitivity of the qPCR assay and thus lead to false-negative results in clinical samples with low amounts of target pathogen. Furthermore, it was reported that mismatches in the probe binding site are more critical to the sensitivity of the qPCR assay than the primer binding site [37,38,39,40]. Considering that 3 mismatched bases were identified in the probe binding site for the NP gene sequences obtained from 11 discordant clinical samples (Figure 2), these false-negative results from the original Schuele’s qRT-PCR are thought to be caused by the incompatibility of the probe with the sequences of some Korean field strains (Figure 2). Therefore, it is necessary to redesign the probe and/or primers of the assay to improve the diagnostic performance of PPIV1 in Korea.

The detection rates of PPIV1 and PPIV5 were different when using the tested clinical samples in this study. The detection rate of PPIV1 was higher in oral fluids (50.0%) than in the lungs (4.2%) and sera (0%) (Table 4), which was consistent with previous reports [4,6,7,9]. A recent experimental challenge study showed that high levels of PPIV1 RNAs were detected in nasal swabs and oral fluids, but not in the serum samples of challenged pigs, indicating that PPIV1 infection may be preliminarily confined to the upper and lower respiratory tracts where it does not produce viremia [41]. Therefore, it is recommended that upper respiratory tract samples, including nasal swabs and oral fluids, be used, as they are more suitable for the clinical analysis and diagnosis of PPIV1 in suspected pig herds. Unlike PPIV1, the detection rate of PPIV5 was relatively low, and only three samples (two lungs and one oral fluid) out of the four hundred and forty-one tested were found to be PPIV5-positive using both the new dqRT-PCR assay and the previous Dong’s qRT-PCR assay. The low detection rate for PPIV5 RNAs (0.7%) was unexpected considering that the serological prevalence of PPIV5 in Korean pig farms was high at 93.8% [19]. However, these results were consistent with a previous experimental challenge study, in which detectable viremia was not produced in SPF piglets challenged with the PPIV5 isolate throughout the study and viral RNAs were not detected in the nasal secretions and lung tissues of the pigs [19]. Nevertheless, given that PPIV5 has been frequently detected or isolated in the lungs, lymph nodes, and intestinal tissues in other field studies [11,13,18], further investigation is required to elucidate the pathogenesis and epidemiology of this viral infection in pig herds through expanded viral testing of a diverse array of clinical pig samples, including intestinal samples.

The farm-level prevalence of PPIV1 was determined to be 11.2%, which was higher than that of Hungary (4.5%, 1/22) but lower than that of Germany (19.2%, 5/26), the USA (81%, 13/16), Poland (76.7%, 23/30), and Chile (100%, 6/6) [2,3,4,8,9,41]. There have been few reports about the farm-level prevalence of PPIV5 using qRT-PCR methods. In this study, the farm-level prevalence of PPIV5 was determined to be 1.1% (3/278) when using dqRT-PCR, which was lower than the previously reported serological prevalence of PPIV5 in Korea (100%, 10/10) [19], indicating that the prevalence of PPIV5 may be underestimated by the qRT-PCR method and that additional diagnostic methods, such as serological methods, will be required to accurately investigate the prevalence of PPIV5 infections.

Recent studies showed that the pathogenic spectrum of PIV5 has been expanded to enteric disease in swine [13,18] and provided evidence that co-infections of PPIVs and other respiratory pathogens may be involved in the pathogenesis of the porcine respiratory disease complex (PRDC) [9]. In this respect, this study had a limitation in that it did not investigate co-infection with PPIVs and other swine respiratory pathogens or viral infection in enteric samples, and further studies are required to elucidate the pathogenesis and epidemiology of the PPIVs associated with PRDC and enteric disease in the field. Furthermore, we found evidence that genetically distinct groups of PPIV1 may exist in Korean pig herds based on the phylogenetic analysis of the partial NP gene sequences in this study, which is inconsistent with a recent Korean study which found that all three Korean PPIV1 strains are grouped into lineage 1 based on the complete genomic sequences of the virus [7]. Therefore, further studies are also required to characterize the genetic diversity of PPIV1 strains in Korea.

## 5. Conclusions

In conclusion, a sensitive and specific dqRT-PCR assay for the differential detection of PPIV1 and PPIV5 has been successfully developed and was used to investigate the prevalence and co-infection status of PPIV1 and PPIV5 in Korean pig farms. The assay was found to have a high sensitivity and specificity and quantitative capabilities. It is thus a promising diagnostic tool for PPIV1 and PPIV5 infections in suspected pig herds and will be useful for etiological diagnosis, epidemiological studies, and the control of PPIV infections.

## Figures and Tables

**Table 1:** Specificity of duplex real-time reverse transcription polymerase chain reaction using PPIV1- and PPIV5-specific primers and probe sets.

Pathogen ^a^	Strain	Source ^b^	Amplification of Target Gene	
			PPIV1 (FAM)	PPIV5 (Cy5)
PPIV1	KPPIV1-2201	ADIC	+	-
PPIV5	KPPIV5-2201	ADIC	-	+
PCV2	PCK0201	ADIC	-	-
PCV3	PCK3-1701	ADIC	-	-
PRRSV-1	Lelystad virus	APQA	-	-
PRRSV-2	LMY	APQA	-	-
SIV	VDS1	APQA	-	-
CSFV	LOM	APQA	-	-
PPV	NADL-2	APQA	-	-
ST cell	-	ADIC	-	-
PK-15 cell	-	ADIC	-	-

^a^ PPIV, porcine parainfluenza virus; PCV, porcine circovirus; PRRSV, porcine reproductive and respiratory syndrome virus; SIV, swine influenza virus; CSFV, classical swine fever virus; PPV, porcine parvovirus. ^b^ APQA, Animal and Plant Quarantine Agency, Korea; ADIC, Animal Disease Intervention Center, Kyungpook National University, Korea.

**Table 2:** Primers and probes used to detect porcine parainfluenza virus 1 (PPIV1) and PPIV5.

Method	Primers /Probe	Sequence (5′–3′) ^a^	Tm (°C )	Genome Position ^b^	Reference
dqRT-PCR for PPIV1	P1-NF	ATAGAGGAGGTGGTGGTG	59.8	178–195	This study
	P1-NR	GTGTTAA**R**AACATGAGTGCTAT	58.4	276–297	
	P1-NP	FAM-TTATACCTGGACAAAAGAACACCGT-BHQ1	64.4	199–233	
dqRT-PCR for PPIV5	P5-NF	CAACAGGGTGCAGTTGA	59.4	1210–1226	This study
	P5-NR	GGTCAATTT**R**GCAAGTGTATT	58.3	1282–1302	
	P5-NP	Cy5-TCTCGGTCTAACTCAAGCCGAACGC-BHQ3	69.2	1245–1269	
qRT-PCR for PPIV1	F	GCCAAAATGGCAGGGTT**R**TT	63.4	114–133	[8]
	R	GCACCACCACCTCCTCTATT	62.9	177–196	
	P	FAM-TGCTCTCACTCCTTTTAGAACTAAATGTG-BHQ1	65	146–174	
qRT-PCR for PPIV5	F	GATCATTCCGCTTAATCCCC	60.3	425–444	[26]
	R	TTCTGCAAGTGCAGCATAGG	62.7	482–501	
	P	FAM-TCGTTCAGGTATGAGCCGTGGA-BHQ1	67	450–471	

^a^ Bold text in the sequences of P1-NR (dqRT-PCR for PPIV1), P5-NR (dqRT-PCR for PPIV5), and R (qRT-PCR for PPIV1) indicates a degenerate base: R, A, or G. FAM, 6-carboxyfluorescein; BHQ1, Black Hole Quencher 1. Cy5, 6-tetramethylindo(di)-carbocyanines 5; BHQ3, Black Hole Quencher 3. ^b^ Locations of the sequences for the PPIV1- and PPIV5-specific primers and probes for the duplex real-time reverse transcription polymerase chain reaction (dqRT-PCR) and reference qRT-PCR assays were derived from the complete genome sequences of the Korean PPIV1 KPPIV1-2201 and PPIV5 KPPIV5-2201 strains (GenBank accession numbers ON457669 and OP734281), respectively.

**Table 3:** Intra- and inter-assay coefficients of variation for the duplex real-time reverse transcription polymerase chain reaction (dqRT-PCR).

Target Pathogen	Dilution (Copies/Reaction)	Intra-Assay			Inter-Assay		
		Mean	SD	CV (%)	Mean	SD	CV (%)
PPIV1	High (10^6^)	19.28	0.06	0.32	19.37	0.13	0.67
	Medium (10^4^)	25.98	0.1	0.4	26.11	0.17	0.66
	Low (10^2^)	32.98	0.03	0.09	33.14	0.17	0.53
PPIV5	High (10^6^)	18.60	0.1	0.55	18.72	0.15	0.8
	Medium (10^4^)	25.08	0.09	0.34	25.19	0.14	0.57
	Low (10^2^)	31.68	0.09	0.29	32.04	0.46	1.44

The mean value, standard deviation (SD), and coefficient of variation (CV) were determined based on the Ct values from the dqRT-PCR.

**Table 4:** Comparison of duplex real-time reverse transcription polymerase chain reaction (dqRT-PCR) and previously reported monoplex qRT-PCR assays for porcine parainfluenza virus 1 (PPIV1) and PPIV5 in clinical samples.

Sample	No. of Tested	No. of Positive Agreements with the dqRT-PCR Assay (%)		No. of Positive Agreements with the Previous qRT-PCR Assays (%)	
		PPIV1	PPIV5	PPIV1	PPIV5
Lungs	168	7 (4.2)	2 (1.2)	6 (3.6)	2 (1.2)
Oral fluids	120	60 (50.0)	1 (0.8) ^a^	42 (35.0)	1 (0.8) ^a^
Sera	153	0	0)	0	0
Total	441	67 (15.2)	3 (0.7)	48 (10.9)	3 (0.7)

^a^ PPIV1 and PPIV5 RNAs were co-amplified in the oral fluid sample using the dqRT-PCR assay. The number of positive, negative, and overall percent agreements for the developed dqRT-PCR were compared with those of Schuele’s qRT-PCR for PPIV1 or Dong’s qRT-PCR for PPIV5, and were 100% (48/48), 95.2% (374/393), and 95.7% (422/441), or 100% (3/3), 100.0% (438/438), and 100% (441/441), respectively. The calculated kappa coefficient values (95% confidence interval) between the dqRT-PCR and previous qRT-PCR for PPIV1 [8] and PPIV5 [26] were 0.81 (0.67–0.95) and 1.0, respectively.

**Table 5:** Diagnostic results of the duplex real-time reverse transcription polymerase chain reaction (dqRT-PCR) and previously reported monoplex qRT-PCR assay for porcine parainfluenza virus 1 (PPIV1) in 19 discordant and 6 concordant clinical samples.

Sample ^a^	Sample Code	Sample Type	Assay Results (Ct Value) ^b^			Sequencing
			dqRT-PCR	qRT-PCR with Probe	qRT-PCR without Probe	
D1	JB1	Oral fluid	32.45	No Ct value	29.13	Yes
D2	JB2	Oral fluid	31.32	No Ct value	28.51	Yes
D3	JB4	Oral fluid	32.09	No Ct value	30.54	Yes
D4	JB15	Oral fluid	35.09	No Ct value	32.65	No
D5	JB33	Oral fluid	34.68	No Ct value	31.59	No
D6	JB34	Oral fluid	36.59	No Ct value	33.92	No
D7	JB47	Oral fluid	35.17	No Ct value	32.41	No
D8	JB53	Oral fluid	33.09	No Ct value	30.14	No
D9	JB54	Oral fluid	33.73	No Ct value	30.03	No
D10	JB79	Oral fluid	30.67	No Ct value	29.24	Yes
D11	JB80	Oral fluid	35.39	No Ct value	32.98	No
D12	JB81	Oral fluid	26.52	No Ct value	25.4	Yes
D13	JB82	Oral fluid	31.18	No Ct value	28.98	Yes
D14	KK36	Oral fluid	27.22	No Ct value	26.14	Yes
D15	KK38	Oral fluid	28.04	No Ct value	27.71	Yes
D16	KK40	Oral fluid	28.26	No Ct value	30.45	Yes
D17	KK42	Oral fluid	27.18	No Ct value	25.02	Yes
D18	KK44	Oral fluid	35.09	No Ct value	33.09	No
D19	P22-013	Lung	27.41	No Ct value	26.55	Yes
C1	JB23	Oral fluid	27.87	31.81	Not Tested	Yes
C2	JB72	Oral fluid	28.01	28.94	Not Tested	Yes
C3	JB74	Oral fluid	27.02	28.13	Not Tested	Yes
C4	KK46	Oral fluid	28.95	31.31	Not Tested	Yes
C5	KK47	Oral fluid	28.27	29.95	Not Tested	Yes
C6	KK177	Oral fluid	30.85	35.76	Not Tested	Yes

^a^ D or C indicate discordant or concordant samples, respectively. ^b^ The qRT-PCR with probe was conducted with a target gene-specific probe as previously described [8], whereas the qRT-PCR without probe was conducted by adding the intercalating dye, SYBR Green, instead of removing the probe from the reaction.

**Table 6:** Prevalence and the co-infection status for porcine parainfluenza virus 1 (PPIV1) and PPIV5 determined using the duplex real-time reverse transcription polymerase chain reaction (dqRT-PCR) assay.

Pathogen	Farm-Level Prevalence			Pig-Level Prevalence		
	No. of Tested	No. of Positive	%	No. of Tested	No. of Positive	%
PPIV1	278	31	11.2	441	67	15.2
PPIV5	278	3	1.1	441	3	0.7
PPIV1 & 5	278	1	0.4	441	1	0.2

## Data Availability

Not applicable.

## Supplementary Materials

The following supporting information can be downloaded at: https://www.mdpi.com/article/10.3390/ani13040598/s1, Table S1: Sequences of porcine parainfluenza virus 1 used for designing primers and probes; Table S2: Sequences of parainfluenza virus 5 strains from different hosts used for designing primers and probes; Figure S1: Alignment of designed primers and probes sequences with nucleocapsid protein (NP) gene sequences of porcine parainfluenza virus 1 (PPIV1) strains; Figure S2: Alignment of designed primers and probe sequences with nucleocapsid protein (NP) gene sequences of porcine parainfluenza virus 5 (PPIV5) strains.
