# Supplementary material for: Molecular Detection of Porcine Parainfluenza Viruses 1 and 5 Using a Newly Developed Duplex Real-Time RT-PCR in South Korea
Source: Animals (Basel). 2023 Feb 8;13(4):598. doi: 10.3390/ani13040598 (PMC9951646; doi:10.3390/ani13040598)
Supplement: Supplementary file 1 [file animals-13-00598-s001.zip › animals-2180627-supplementary/Supplementary Materials - Figure S1.pdf]

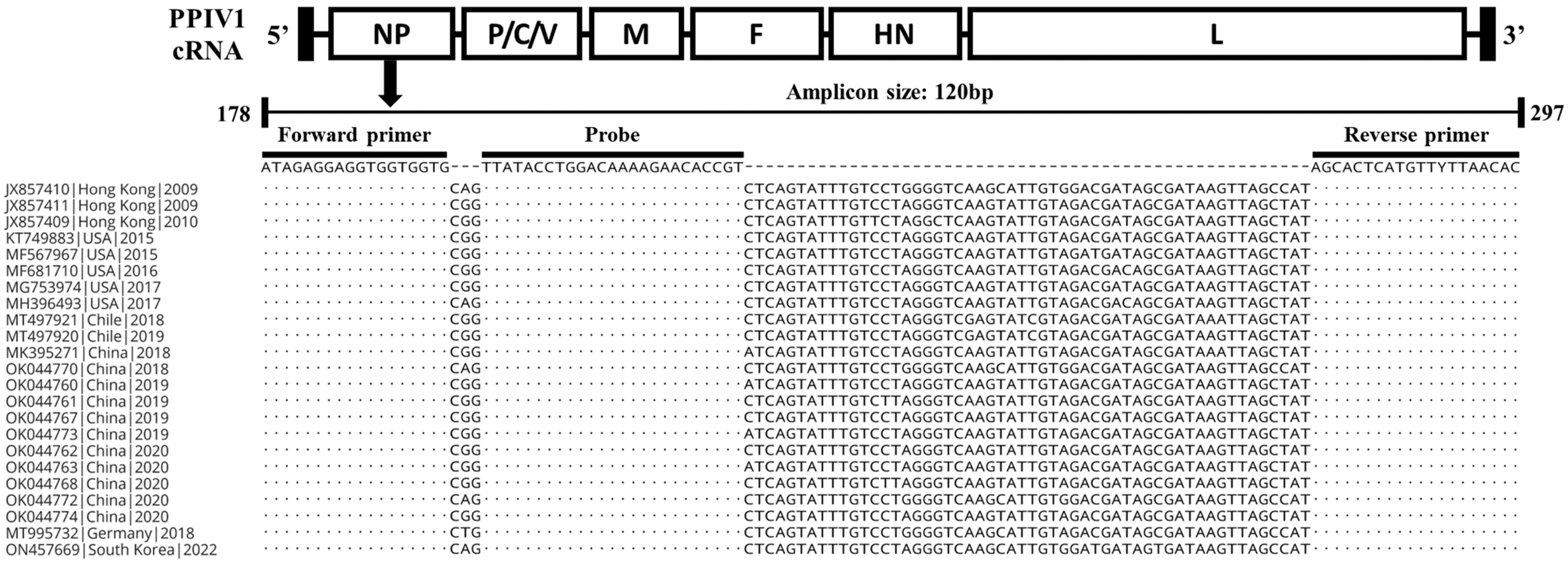

Figure S1. Alignment of designed primers and probes sequences with nucleocapsid protein (NP) gene sequences of porcine parainfluenza virus 1 (PPIV1) strains. A dot indicates the same base, and a letter indicates a different base between primers/probes and targeted NP genes sequences of PPIV1 strains. The sequences were aligned in Geneious version 2022.0.2 via MAFFT.
