# Supplementary material for: Molecular Detection of Porcine Parainfluenza Viruses 1 and 5 Using a Newly Developed Duplex Real-Time RT-PCR in South Korea
Source: Animals (Basel). 2023 Feb 8;13(4):598. doi: 10.3390/ani13040598 (PMC9951646; doi:10.3390/ani13040598)
Supplement: Supplementary file 1 [file animals-13-00598-s001.zip › animals-2180627-supplementary/Supplementary Materials - Table S1.pdf]

Table S1. Sequences of porcine parainfluenza virus 1 used for designing primers and probes.

No.	Strain	Country	Year	GenBank No.
1	S033N	Hong Kong	2009	JX857410
2	S119N	Hong Kong	2009	JX857411
3	S206N	Hong Kong	2010	JX857409
4	3103-1	USA	2015	KT749883
5	15TOSU0582	USA	2015	MF567967
6	USA/MN25890NS/2016	USA	2016	MF681710
7	USA/IA84915LG/2017	USA	2017	MG753974
8	KS17-258	USA	2017	MH396493
9	Chile/VN14014564/2019	Chile	2019	MT497920
10	Chile/VN14012673/2018	Chile	2018	MT497921
11	gd2018	China	2018	MK395271
12	ZJ03	China	2019	OK044760
13	ZJ04	China	2019	OK044761
14	ZJ05	China	2020	OK044762
15	ZJ06	China	2020	OK044763
16	ZJ10	China	2019	OK044767
17	ZJ11	China	2020	OK044768
18	ZJ13	China	2018	OK044770
19	ZJ16	China	2020	OK044772
20	ZJ17	China	2019	OK044773
21	ZJ18	China	2020	OK044774
22	GER/LEHJN/2018	Germany	2018	MT995732
23	KPPIV1-2201	South Korea	2022	KT749883
