# Supplementary material for: Molecular Detection of Porcine Parainfluenza Viruses 1 and 5 Using a Newly Developed Duplex Real-Time RT-PCR in South Korea
Source: Animals (Basel). 2023 Feb 8;13(4):598. doi: 10.3390/ani13040598 (PMC9951646; doi:10.3390/ani13040598)
Supplement: Supplementary file 1 [file animals-13-00598-s001.zip › animals-2180627-supplementary/Supplementary Materials - Table S2.pdf]

Table S2. Sequences of parainfluenza virus 5 strains from different hosts used for designing primers and probes.

No.	Host	Strain	Country	Year	GenBank No.
1	Pig	SER	Germany	1998	JQ743328
2	Pig	KNU-11	South Korea	2011	KC852177
3	Pig	T220	South Korea	2016	MK423232
4	Pig	T263	South Korea	2016	MK423233
5	Pig	T361	South Korea	2016	MK423234
6	Pig	T398	South Korea	2016	MK423235
7	Pig	T399	South Korea	2016	MK423236
8	Pig	T434	South Korea	2016	MK423237
9	Pig	Carina (PF-2)	South Korea	2017	MF170888
10	Pig	Rigel (PF-1)	South Korea	2017	MF170889
11	Pig	M32	South Korea	2017	MK423238
12	Pig	M129	South Korea	2017	MK423239
13	Pig	M197	South Korea	2017	MK423240
14	Pig	M293	South Korea	2017	MK423241
15	Pig	N99	South Korea	2018	MK423242
16	Pig	N163	South Korea	2018	MK423243
17	Pig	KPPIV5-2201	South Korea	2019	OP734281
18	Pig	SH/2015/1202	China	2015	MK028670
19	Pig	HLJ2015/DP1-1/PIV5	China	2015	MT890696
20	Pig	HLJ2015/DP2-1/PIV5	China	2015	MT890697
21	Pig	SH/2015/122/PIV5	China	2015	MT890698
22	Pig	HuB/YC/2015/PIV5	China	2015	MT890699
23	Pig	JX/2015/1221/PIV5	China	2015	MT890700
24	Pig	HLJ	China	2020	OK505006
25	Pig	IND/MZ/3013789	India	2017	MW273368
26	Pig	IND/MZ/3013814	India	2017	MW273369
27	Dog	CPI-	USA	1980	JQ743320
28	Dog	CPI+	USA	1980	JQ743321
29	Dog	FA	China	2007	EF543648
30	Dog	FC	China	2007	EF546391
31	Dog	CPIV-HeN0718	China	2015	KY114804
32	Dog	CC-14	China	2015	KP893891
33	Dog	D277	South Korea	2008	KC237065
34	Dog	1168-1	South Korea	2009	KC237064
35	Dog	08-1990	South Korea	2009	KC237063
36	Dog	78524	United Kingdom	2012	JQ743319
37	Dog	H221	United Kingdom	2012	JQ743323
38	Dog	CU-D133	Thailand	2016	MT603999
39	Dog	CU-D151	Thailand	2016	MT604000
40	Dog	CU-D20804	Thailand	2018	MT604001
41	Cattle	CH18-MMH	Switzerland	1998	MN735204
42	Cattle	PV5-BC14	China	2014	KM067467
43	Monkey	W3A	USA	1964	JQ743318
44	Lesser panda	ZJQ-221	China	2015	KX100034
45	Tiger	PIV5-SR	China	2015	KY685075
46	Pangolin	PIV5-GD18	China	2017	MG921602

Table S2. *Cont.*

No.	Host	Strain	Country	Year	GenBank No.
47	Pangolin	CAN	China	2018	MH362816
48	Horse	XJ033	China	2018	MN604146
49	Tiger	HMZ	China	2018	MH370862
50	Snake	China/HB01/2019	China	2019	MT124463
51	Tick	Tick/Heilongjiang/2019	China	2019	MW051776
52	Human	RQ	United Kingdom	1976	JQ743327
53	Human	DEN	United Kingdom	1980	JQ743322
54	Human	LN	United Kingdom	1980	JQ743324
55	Human	MEL	United Kingdom	1980	JQ743325
56	Human	MIL	United Kingdom	1980	JQ743326
57	Human	AGS	USA	1983	KX060176
58	Human	L	China	2017	MT160087
